# Yet Another Potential Age-Old Nonpharmaceutical Intervention

**DOI:** 10.3201/eid2711.AC2711

**Published:** 2021-11

**Authors:** Kathleen Gensheimer, Byron Breedlove

**Affiliations:** Food and Drug Administration, College Park, Maryland, USA (K. Gensheimer);; Centers for Disease Control and Prevention, Atlanta, Georgia, USA (B. Breedlove)

**Keywords:** art science connection, emerging infectious diseases, art and medicine, about the cover, public health, vaccines, antibiotics, antiviral drugs, respiratory pathogens, respiratory infections, pneumonia, influenza, influenza A and B, parainfluenza, metapneumovirus, measles, respiratory syncytial virus, Hanging the Laundry Out to Dry, Berthe Morisot, Yet Another Potential Age Old Nonpharmaceutical Intervention, impressionism¸ nonpharmaceutical interventions

**Figure:**
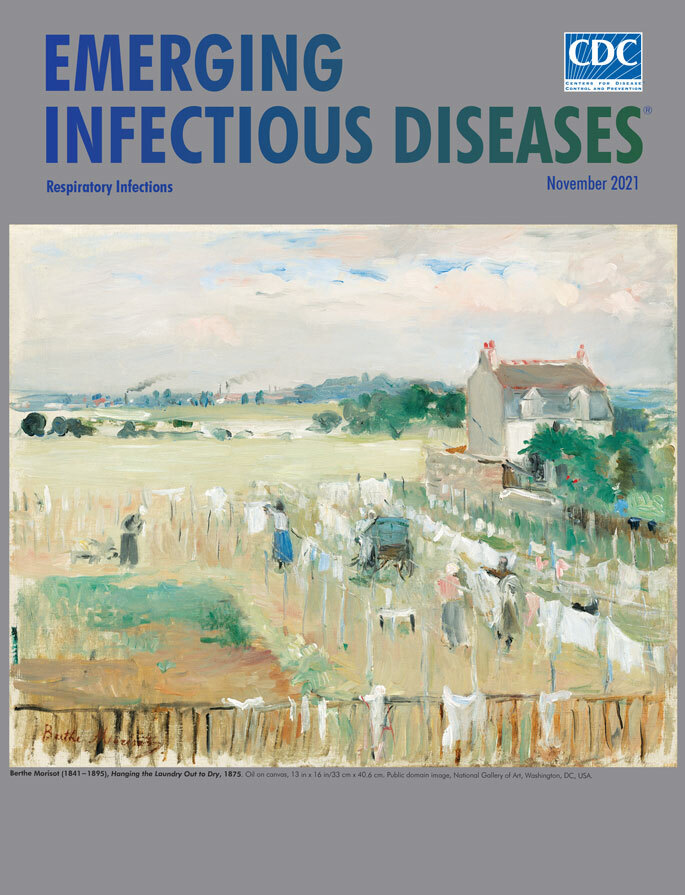
**Berthe Morisot (1841−1895), Hanging the Laundry Out to Dry, 1875.** Oil on canvas, 13 in x 16 in/33 cm x 40.6 cm. Public domain image, National Gallery of Art, Washington, DC, USA.

In 1874, members of the *Société Anonyme des Artistes-Peintres, Sculpteurs, Graveurs* staged an exhibition of their work in the highly esteemed Salon de Paris, launching a movement now identified as Impressionism. Art historian Margaret Samu writes, “Their work is recognized today for its modernity, embodied in its rejection of established styles, its incorporation of new technology and ideas, and its depiction of modern life.” But this nascent art form was not universally applauded. Art and culture critic Jason Farago notes, “The movement’s name was originally a critic’s insult. ‘Impressionist’ came from a venomous review of an 1874 exhibition of paintings by Monet, Renoir, Degas, Pissarro—and one woman.” A second exhibition in the spring of 1876 in Paris induced more mixed reactions; one detractor described its participants as "five or six lunatics, one of which is a woman.”

That woman, Berthe Morisot, became a leading figure of the Impressionist artistic movement of the 19th century and is perhaps the most underrated Impressionist. The granddaughter of the Rococo painter Jean-Honoré Fragonard, Morisot decided at an early age to become an artist. From 1862 to 1868, she worked under the guidance of landscape artist Camille Corot. Morisot exhibited paintings at the Salon de Paris from 1864 through 1874, when, in support of the burgeoning Impressionist movement, she vowed to never again show her paintings in the officially sanctioned forum. In 1868, Morisot developed a working friendship with French modernist painter Édouard Manet. Manet had a liberating effect on her work, and she in turn aroused his interest in outdoor painting. In 1874, she married Manet’s younger brother, Eugène, a writer and painter.

Columnist Tessa Solomon explains that “. . . Morisot’s gender also played a role in how she was perceived. Writers in her day used terms like ‘flirtatious’ and ‘charming’ to describe her work; neither were labels given to the paintings of Claude Monet, Pierre-Auguste Renoir, and others.” Even today, sexist undertones surface in the ways in which Morisot is discussed. In 2018, when the Barnes Foundation in Philadelphia mounted the first US retrospective devoted to her, it was subtitled “Woman Impressionist.” “Imagine a parallel case, say, ‘Georges Braque: Man Cubist,'” quipped art critic Peter Schjeldahl. 

Social conventions of the day kept Morisot from pursuing the same subject matter as her male counterparts, such as Monet and Renoir, who often painted popular sites of leisure around Paris. Because Morisot liked to paint outdoors―and frequenting such sites without a chaperone would have invited scandal―she instead depicted domestic scenes, landscapes, and portraits, stating, “It is important to express oneself, provided the feelings are real and are taken from your own experience.” Like Manet, she portrayed contemporary life, taking inspiration from quotidian life. Much of her work focused on the lives of women in French society, and this month’s cover image, *Hanging the Laundry Out to Dry*, is an example of her *plein-air* painting; that is, painting outdoors, which better captures the appearance of light and weather conditions. 

Morisot depicts several women hanging the washing to dry on a windy day. Clothing hangs off almost everything conceivable object in the garden. In the background, trees dot the countryside, perhaps marking the edges of the property, and steam trains travel across the horizon. Clumps of billowing clouds race across the canvas, revealing glimpses of blue sky. In the foreground, a wood fence that parallels the distant horizon is also draped with laundry. By relying on flickering brushstrokes and a light palette, Morisot succeeds in briskly conveying a scene, not fixating on accuracy or detail.

Art historian Aleid Ford observes, “Figures and features of the scene are roughed-in rapidly. Perspective makes quick sense of the scene but Morisot doesn’t dither with sharp or acute detail. Rather, she seems to scrub the view clean with a bleached-out palette of pastels, anchoring the lot with that spindled fence along the front.” The large house and extended grounds suggest that a wealthy family lives there and employs a number of workers to handle daily chores such as laundry. According to exhibition notes from the Barnes Foundation, “Working women are a recurring subject in Morisot’s painting. The cooks, maids, and servants employed by upper-middle-class households in the late 19th century were as much a part of Morisot’s daily life as her family and friends.”

A prolific artist, Morisot never enjoyed great commercial success despite having attained significant critical recognition during her lifetime. In 1895, Morisot’s daughter Julia was ill with pneumonia, and although Julia recovered, Morisot succumbed to the disease while caring for her daughter and died at the age of 54. 

During Morisot’s lifetime, today’s vaccines and antimicrobials that can prevent or treat pneumonia did not exist. Even with prevention and treatment availability, pneumonia continues to affect hundreds of millions of people, old and young, around the globe. Many cases of pneumonia are caused by transmissible pathogens associated with outbreaks of disease, including influenza A and B virus, parainfluenza, metapneumovirus, measles virus, and respiratory syncytial virus. Of particular importance is the ongoing coronavirus disease pandemic, approaching its second year of circulating globally. Its mitigation requires both vaccines and nonpharmaceutical interventions.

Respiratory infections caused by transmissible pathogens can also be mitigated by wearing masks, maintaining physical distance, practicing hand and face hygiene, cleansing surfaces, avoiding crowds, and increasingly engaging in outdoor activities as feasible. Morisot’s portrayal of women working outdoors in the fresh air and sunshine may be a picture of days past, but, nonetheless, it now also conveys a modern message, showing how nonpharmaceutical interventions can help prevent the transmission of respiratory diseases.
